# Prevalence and risk factors of coccidiosis in calves from Veracruz, México

**DOI:** 10.1590/S1984-29612022043

**Published:** 2022-08-08

**Authors:** Angélica Olivares-Muñoz, Miguel Angel Alonso-Díaz, Dora Romero-Salas, Anabel Cruz-Romero, Manuel Barrientos-Morales, Juan Manuel Pinos-Rodríguez

**Affiliations:** 1 Facultad de Medicina Veterinaria y Zootecnia, Universidad Veracruzana, Veracruz, México; 2 Centro de Enseñanza, Investigación y Extensión en Ganadería Tropical, Universidad Nacional Autónoma de México, Veracruz, México

**Keywords:** Cattle, *Eimeria* spp., prevalence, risk factors, Veracruz, Bovinos, *Eimeria* spp., prevalência, fatores de risco, Veracruz

## Abstract

The objectives of the present study were: (1) to determine the prevalence of *Eimeria* spp. sporulated oocysts in calves from 26 Municipalities in the Central Zone of the State of Veracruz, Mexico, (2) to identify the *Eimeria* spp. infecting calves, and (3) to identify the risk factors associated with the presence of *Eimeria* spp. in tropical cattle. A total of 930 individual fecal samples were analyzed by using the McMaster technique; then, oocysts were maintained in 2.5% potassium dichromate to allow sporulation. The general prevalence of calves with *Eimeria* spp. oocysts was 39.7% (370/930). Of a total of 10 identified species, *Eimeria canadensis* was the most observed, followed by *Eimeria bovis* and *Eimeria zuernii*. The statistical analysis showed an association between the age of the calves (5 to 9 months), the presence of other animals, the physiographic regions and the restricted type of husbandry with the presence of *Eimeria* spp. in calves (P<0.05). Protective risk factors, such as: routine coprological analysis was also associated with a decrease in infection. These data demonstrate the presence of coccidia in cattle from the State of Veracruz, additionally to the necessary measures that must be taken to control this parasitosis.

## Resumo

Os objetivos do presente estudo foram: (1) determinar a prevalência de oocistos esporulados de *Eimeria* spp. em bezerros de 26 municípios na zona central do estado de Veracruz, México; (2) identificar as espécies de *Eimeria* que infectam bezerros; e (3) identificar os factores de risco associados à presença de *Eimeria* spp. em bovinos de região tropical. Um total de 930 amostras individuais de fezes foi analisado usando-se a técnica McMaster. Depois oocistos foram mantidos em 2,5% de dicromato de potássio para esporulação. A prevalência geral de animais com oocistos de *Eimeria* spp. foi de 39,7% (370/930). De um total de 10 espécies identificadas, *Eimeria canadensis* foi a mais observada, seguida de *Eimeria bovis e Eimeria zuernii*. A análise estatística indicou uma associação entre a idade dos bezerros (5 a 9 meses), a presença de outros animais, as regiões fisiográficas e o tipo restrito de criação com a presença de *Eimeria* spp. em bezerros (P<0,05). Os fatores de risco protetores, tais como a análise coprológica de rotina, foram também associados a uma diminuição da infecção. Esses dados indicaram a presença de coccídios em bovinos do estado de Veracruz, além das medidas necessárias que devem ser tomadas para controlar essa parasitose.

## Introduction

Bovine coccidiosis is a gastrointestinal disease associated with apicomplexans of the genus *Eimeria*, where at least 21 species have been reported in cattle around the world (Lee et al., 2018). Coccidiosis is one of the most economically important among diseases in the livestock industry that causes both clinical and subclinical losses, especially in young cattle (Lopez-Osorio et al., 2018). Clinical infection may result in diarrhea, anorexia, weakness, dehydration, and occasional deaths (Tomczuk et al., 2015); whereas subclinical diseases are defined by weight loss, reduced weight gain, and loss of appetite, resulting in potentially long-lasting effects (Fitzgerald, 1980). Subclinical cases are more common and are proposed to quietly disrupt intestinal physiology, thereby resulting in high feed conversion (Ekawasti et al., 2022). The control of coccidiosis in the herd consists of reducing the parasitic load by improving the hygienic measures of the cattle habitat, as well as stress management and colostrum feeding, among other activities (Bangoura & Bardsley, 2020); and/or the use of chemical drugs to inhibit the life cycle as well as eliminate the etiological agents responsible for the disease (Philippe et al., 2014).

The presence of coccidiosis on farms and in individual animals can be influenced by a wide group of intrinsic and extrinsic factors (Tomczuk et al., 2015). Perhaps the risk factor most associated with coccidiosis in cattle has been the age of the host. Young animals (less than 1 year old) are more susceptible to coccidiosis, as well as the presence of animals of different ages that are housed in the same place (Hermosilla et al., 2012). Alcala-Canto et al. (2020) mentioned that macroenvironmental variables such as temperature and rainfall influence the presence of *Eimeria* spp. in cattle and, for some species, different factors such as: rearing system, type of installation, farm size, seasonality and altitude affect the occurrence of coccidiosis. The presence of *Eimeria* spp. is attributed to subtropical zones with humid - sub-humid climates, the size of the UPB, as well as the feeding type of the animals. Although in Mexico, the State of Veracruz has climatic and management factors that favor the occurrence of *Eimeria* spp. in cattle, studies at farm level are scarce (INEGI, 2018). The presence of coccidiosis in livestock production units in Mexico represents economic losses of up to $23.7 million dollars per year (Rodríguez-Vivas et al., 2017).

Epidemiological information on the presence of *Eimeria* spp. in tropical livestock farms is a basic step in building an efficient coccidiosis control program and improving health and well-being. Likewise, updated information is necessary on the species of *Eimeria* that are circulating in Veracruz. Therefore, the objectives of the present study were: (1) to determine the prevalence of *Eimeria* spp. oocysts in calves from 26 Municipalities in the central zone of the State of Veracruz, Mexico, (2) to identify *Eimeria* spp. infecting calves, and (3) to identify the risk factors associated with their presence.

## Material and Methods

### Ethical considerations

The work was approved by the Bioethics Committee of Faculty of Veterinary Medicine and Zootechnics, University of Veracruz, under the protocol n. 007/21.

### Study area and sample size

A cross-sectional study was carried out on 62 production units through 26 Municipalities of the physiographic region of Sotavento, Las Montañas, Papaloapan, Capital and Nautla (from August 2020 to April 2021). These areas belong to the central region of the State of Veracruz, where there is a humid tropical climate with an average annual temperature of 23.4 ± 0.5, an annual rainfall of 1,991 ± 392 mm, and a mean relative humidity (RH) of 85% (INEGI, 2018).

The sample size (n = 367) was calculated using an expected prevalence of *Eimeria* spp. of 60% (Alcala-Canto et al., 2020), an animal census of 91,589 calves (México, 2018), a confidence level of 95% and statistical error of five (Thrusfield, 1995). The selection of cattle farms and animals were for convenience (Thrusfield, 1995). Due to a greater participation of cattle producers in the study, as well as a greater number of animals within each cattle production unit, the sample size (n) increased to 930 calves.

### Animals and sample collection

For each livestock farm, young animals and calves were selected and assigned to three age groups: ≥1 – 4, 5 – 9 and 10 – 13 months (with or without the presence of clinical signs of coccidiosis). Calves dewormed 15 days before the sample collection were excluded from the study.

At least five grams of feces were collected directly from the rectum of each calve in order to perform the parasitological tests mentioned below. The feces were identified by number or name, age, and sex and transported in a plastic cooler (4–5^◦^C) to the Parasitology Laboratory of the University of Veracruz, where they were kept in refrigeration at 4º C for 24 hours before analysis.

### Laboratory analysis

The fecal oocyst cou nt per gram of feces (OoPG) was determined using McMaster technique according to Taylor et al. (2016). Positive samples ≥ 500 OoPG were subjected to a sporulation process, using 2.5% potassium dichromate in Petri plates and incubated at room temperature, followed by oxygenation of the samples every 24 hours for ten days (Cruvinel et al., 2018). The oocysts were isolated by flotation in sugar solution (specific gravity 1.27) (Florião et al., 2016), and approximately 10 oocyst per pool from each farm were identified under an optical microscope using a Velab VE-87 (Puebla, Mexico) x 1000 magnification. This identification was done according to the phenotypic characteristics of the sporulated oocysts according to Taylor et al. (2016) (Table 1).

**Table 1:** Description of the sporulated oocyst *Eimeria* species in cattle from the central zone of Veracruz.

**Species**	**Oocyst description**	**Mean size (μm)**
***E. bovis***	Ovoid or subspherical, colourless, and smooth wall with inconspicuous micropyle, no polar granule or oocyst residuum	28 x 20
***E. zuernii***	Subspherical, colourless, with no micropyle or oocyst residuum	18 x 16
***E. alabamensis***	Usually ovoid with a smooth colourless wall with no micropyle, polar body or residuum	19 x 13
***E. auburnensis***	Elongated, ovoid, yellowish‐brown, with smooth or heavily granulated wall with a micropyle and polar granule, but no oocyst residuum	38 x 23
***E. bukidnonensis***	Pear shaped or oval, tapering at one pole, yellowish brown, with a thick, radially striated wall and micropyle. A polar granule may be present but there is no oocyst residuum	49 x 35
***E. canadensis***	Ovoid or ellipsoidal, colorless, or pale yellow, with an inconspicuous micropyle, one or more polar granules and an oocyst residuum	33 x 23
***E. cylindrica***	Elongated cylindrical with a colorless smooth wall, no micropyle, and no oocyst residuum	23 x 12
***E. ellipsoidalis***	Ellipsoidal to slightly ovoid, colorless, with no discernible micropyle, polar granule or oocyst residuum	23 x 16
***E. subspherica***	Round or subspherical, colorless, with no micropyle, polar granule, or oocyst residuum	11 x 10
***E. wyomingensis***	Ovoid, yellowish‐brown, with a thick wall, a wide micropyle but no polar granule or oocyst residuum	40 x 28

### Questionnaire of the Bovine Production Unit

A survey was applied to the managers of the Bovine Production Unit, which contained dichotomous and multiple-choice answers, to obtain information by animal and management practice in the unit. Table 2 shows the variables studied for the analysis of risk factors.

**Table 2:** Variables considered as possible risk factors for the presence of coccidiosis in calves from the central zone of Veracruz.

**Variable**	**Description**	**Term/Answer**
Physiographic region	Municipalities	S, M, P, N, C*
Stocking density	Number of cattle per unit	1-50/ 51-100 / 101-200
Age	Months	1 -4 / 5-9 / 10-13
Gender	Gender	Male / Female
Diarrhea	Presence or absence of signs	Yes/ No
Zootechnical function	Specific zootechnical activity of the Unit	Meat/ Milk / Double purpose
Animal Production system	Farming system	Intensive/ Semi intensive/ Extensive
Other species	Presence or absence of other animal species	Yes / No
Breed	Cattle breed	Zebu / European/ Zebu- European
Body condition	Scale from 1 to 5	Bad / Regular/ Good
Calf rearing	Type of calf rearing	Artificial rearing / Restricted suckling / Permanent suckling
Type of housing	Facilities	Open / Close / Mixed
Type of floor	Flooring	Cemented / Partially/ No cemented
Type of water	Potable / No potable	Yes / No
Puddles	Presence or absence of puddles in the unit	Yes/ No
Cleaning	Stable cleaning	Yes / No
Disinfection	Use of disinfecting agents	Yes/ No
Veterinary doctor	Presence or absence of a veterinarian in the unit	Yes/ No
Coprological studies	Analysis of the fecal samples by the staff	Yes/ No
Deworming	Presence or absence of deworming drugs	Yes/ No

* S = Sotavento; M = Las Montañas; P = Papaloapan; N = Nautla; C= Capital.

### Statistical analysis

Prevalence of cattle parasitized with *Eimeria* spp. was calculated using the following Formula 1:


Prevalence= No. of animals withcoccidiosis / total number of sampled animals (1)

Associations between independent and dependent variables were, first, analyzed using univariate analysis with Chi-square (χ^2^) test with Fisher correction. Posteriorly, all the variables with a significance value of (*p) ≤* 0.20 with χ^2^ test, were entered in a logistic regression model (Stata program Version 14.0, Texas, USA), which provides exact regression estimates, 95% confidence intervals (CI _95%_), odd ratios (*OR*: a measure of association which quantifies the relationship between exposure variables and outcomes), p-values (P), standard error (SE) and a beta value (regression coefficient).

## Results

### Prevalence of *Eimeria* spp.

The overall prevalence of calves with coccidiosis in the central zone of the state of Veracruz was 39.7% (370 / 930; CI_95%_ 36.6 - 42.9). This study revealed a higher prevalence of coccidiosis in the physiographic region of Nautla and Capital with 48.8% and 41.3% (42/87, CI_95%_ 38.0-58.6;104/251 CI_95%_35.5-47.6), respectively; followed by female gender with 39.9% (237/578; CI_95%_ 36.0-44.0) and the presence of diarrhea with 43.0% (96/223; CI_95%_ 36.7 -49.6).

### *Eimeria* species infecting calves

**Figure 1:**
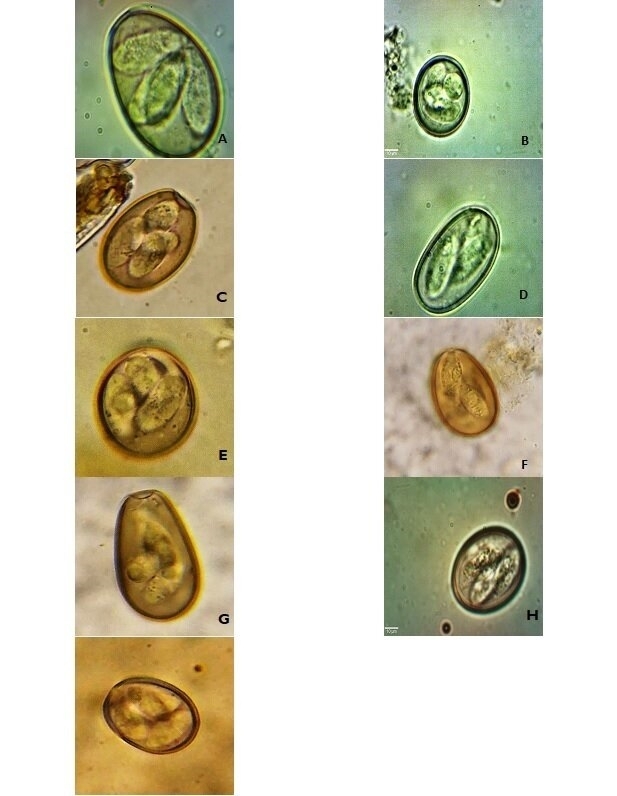
Images from optical microscope of sporulated *Eimeria* species oocyst observed (x 1000 magnification) (A) *E. bovis*, (B) *E. zuernii*, (C) *E. canadensis*, (D) *E. cylindrica*, (E) *E. alabamensis*, (F) *E. wyomingensis*, (G) *E. auburnensis*, (H) *E. ellipsoidalis* and (I) *E. subspherica.*

A total of 10 species of *Eimeria* were identified: *Eimeria canadensis, Eimeria bovis, Eimeria cylindrica, Eimeria auburnensis, Eimeria zuernii, Eimeria ellipsoidalis, Eimeria wyomingensis, Eimeria alabamensis, Eimeria bukidnonensis* and *Eimeria subspherical*. *Eimeria canadensis* was the most observed (25.9%) followed by *E. bovis* (24.2%) and *E. zuernii* (15.7%) (Table 3). Images on sporulated *Eimeria* spp. oocysts are presented in Figure 1. The image of *E. bukidnonensis* was not displayed due to technical problems; but it was identified through the size and characteristics according to Taylor et al. (2016). 

**Table 3:** Frequency and morphological measurements of *Eimeria* spp. identified in the central zone of Veracruz, Mexico.

**Species**	**#Oocyst**	**Frequency (%)**	**CI_95%_**	**Length (µm)**	**Width (µm)**	**Shape index**
*Eimeria canadensis*	127/490	25.9	23.7-31.8	31	20	1.6
*Eimeria bovis*	119/490	24.2	20.7-28.2	28	20	1.4
*Eimeria zuernii*	77/490	15.7	12.7-19.2	22	12	1.8
*Eimeria cylindrica*	53/490	10.8	8.3-13.8	38	26	1.5
*Eimeria ellipsoidalis*	47/490	9.5	7.2-12.5	18	15	1.2
*Eymeria auburnensis*	41/490	8.3	6.2-11.1	16	20	0.8
*Eimeria wyomigensis*	18/490	3.6	2.3-5.7	39	26	1.5
*Eimeria alabamensis*	4/490	0.8	0.3-2.0	20	13	1.5
*Eimeria bukidnonensis*	2/490	0.4	0.11-0.15	48	23	2.0
*Eimeria subspherical*	2/490	0.4	0.11-0.15	14	12	1.1

### Risk factors

Table 4 shows the univariate analysis of the presence of *Eimeria* spp. with the type of variable responses. Risk factors that showed greater association with the occurrence of *Eimeria* spp. in calves were the physiographic region, the age, the presence of other animal species and the type of calf rearing (P<0.05; Table 5). Calves from the region of Nautla had 1.55 greater probability of being infected with *Eimeria* (*OR*1.55; CI_95%_ 1.07-2.26) compared with the Papaloapan region. The 5 - 9-month-old group had a higher number of oocysts compared to 10–13-month-old calves (*OR* 1.49 (CI_95%_ 1.08-2.04; *P=*0.013). Furthermore, the presence of other animal species [*OR* of 1.86 (CI_95%_ 1.31-2.55)] and calves with restricted suckling [*OR* of 2.31 (CI_95%_ 1.37-2.39)] had higher probabilities of being infected with *Eimeria* spp. These data are summarized in Table 5.

**Table 4:** Univariate analysis results (x2) for the identification of possible risk factors associated with calves parasitized with *Eimeria* spp. in the central zone of Veracruz, México.

**Variable**	**Type of response x^2^** ***(p-* value*)***	**Type of fisher response *(p-* value)**
Physiographic region	0.02	*-*
Stocking density	0.37	*-*
Age	0.09	-
Gender	0.88	0.89
Diarrhea	0.25	0.27
Zootechnical function	0.14	-
Animal Production system	0.07	-
Other species	0.06	0.06
Breed	0.008	-
Body condition	0.78	-
Type of rearing	0.02	-
Type of housing	0.86	-
Type of floor	0.38	-
Type of water	0.06	0.07
Puddles	0.5	0.60
Cleaning	0.54	0.58
Disinfection	0.74	0.78
Veterinary doctor	0.13	0.14
Coprological studies	<0.0	<0.0
Deworming	0.50	0.55

**Table 5:** Logistic regression analysis to identify risk factors associated with calves parasitized with *Eimeria* spp. in the central zone of Veracruz, Mexico.

**Variable**	**OR**	**CI _95%_**	***P***	**SE**
Physiographic region				
Sotavento	1 (Ref)			
Montañas	1.56	0.90-2.70	0.107	0.43
Papaloapan	0.50	0.26-0.99	0.05	0.17
Nautla	1.55	1.07-2.26	0.19	0.29
Capital	0.63	0.36-1.10	0.11	0.18
Age (months)				
1 – 4	1(Ref)	-	-	
5 – 9	1.49	1.08-2.04	0.013	0.24
10-13	1.43	0.96-2.12	0.076	0.28
Other species				
No	1 (Ref)			
Yes	1.86	1.31-2.65	0.001	0.33
Type of rearing				
Artificial calf rearing	1 (Ref)	^-^	-	-
Restricted calf suckling	2.31	1.37-3.89	0.002	0.61
Continuous calf suckling	1.5	0.94-2.41	0.086	0.36
Coprological studies				
No	1 (Ref)			
Yes	0.29	0.19-0.45	0.00	0.064

### Protective factors

In this study, we determine that the physiographic region of Papaloapan and the performance of coprological studies in the animal unit should be considered as protective factors, since they obtained OR values of 0.50 (CI_95%_ 0.26-0.99) and 0.29(CI_95%_ 0.19-0.45), respectively.

## Discussion

The first objective of the present study was to determine the prevalence of *Eimeria* spp. oocysts in calves from 26 Municipalities in the center area of the State of Veracruz, México. The overall prevalence of calves with coccidiosis was 39.7%. These results were consistent with the previous ones acquired by Quiroz & Casillas (1971) concerning the prevalence of 38% of the parasite in the south of Veracruz. The State of Veracruz has the ideal conditions that facilitated the presence of *Eimeria* spp. such as: the climate, physiography, and tropical areas, which are considered the best means for the parasite to survive and reproduce (Lucas et al., 2014). This State has tropical and subtropical climates, which facilitate the survival of *Eimeria* spp. in the yards of young animals (INEGI, 2018). However, the prevalence shown in this analysis is below compared with other studies made in México with cattle (60.2%) (Alcala-Canto et al., 2020) and other countries such as Brazil (66%) (Florião et al., 2016), Indonesia (65.4%), (Ekawasti et al., 2022), Iran (63%) (Adinehbeigi et al., 2018) and Colombia (75.6%) (Lopez-Osorio et al., 2020). This variation is most likely attributed to the differences in study design, biotic and abiotic factors and husbandry practices of the study animals in different countries (Tamrat et al., 2020), a high animal density, accumulation of organic material and mix of animals of different ages (Florião et al., 2016). On the other hand, in this study, a high prevalence was observed in the physiographic region of “Nautla” with 48.8%. Nautla represents 3.2% of the cattle volume in the state of Veracruz. It has 12 regions with hydrological basins where a warm humid climate prevails with rains all year round. In addition, it has an annual rainfall of 1,383.00 and a maximum temperature of 40^o^ C. The *Eimeria* spp. shows a significant association with humid and sub-humid areas and high temperatures, in addition to high rainfall that allows the development and abundance of the parasite (Das et al., 2015).

Bovine coccidiosis is a gastrointestinal disease associated with apicomplexans of the genus *Eimeria*, where at least 21 species have been reported in cattle around the world (Lee et al., 2018). The second objective of this study was to be observed *Eimeria* spp. natural infecting calves. From a total of 10 species of *Eimeria* were identified, *E*. *canadensis* was the most observed followed by *E. bovis* and *E. zuernii*. Of the 12 *Eimeria* spp. previously reported for Mexico by Alcala-Canto et al. (2020), *E. cylindrica* and *E. ellipsoidalis* were the most observed in cattle. However, in the same spatial distribution of *Eimeria* species of cattle in Mexico, *E*. *canadensis* was the most encountered species with a frequency of 77.58% in temperate humid climates, but also had a high prevalence in warm semi humid and humid climates with 68.13% and 67.08%, respectively. The presence of *E. canadensis* does not cause significant damage to the intestine of the infected animals, since this species is not considered pathogenic within the pathogenicity classification (Bangoura & Bardsley, 2020), however, their high frequency in this study deserves future epidemiological and clinical studies throughout the year. It is mentioned that economical losses due to subclinical disease even are higher than clinical coccidiosis (Fitzgerald, 1980) because it is more common and can affect the productive and reproductive parameters in cattle (Larsson et al., 2006).

In our study, *E*. *bovis* and *E*. *zuernii* were detected with an important frequency of 24.2% and 15.7%, respectively. This finding agrees with Quiroz & Casillas (1971) who described *E. bovis* as the most frequent in cattle. These results are also consistent with several authors reporting the species *E. bovis* and *E. zuernii* identified more frequently in the fecal samples analyzed (Bruhn et al., 2012; Lee et al., 2018; Hamid et al., 2019; Ola-Fadunsin et al., 2020; Cruvinel et al., 2021). Although mixed infections are commonly observed under natural conditions, only two highly pathogenic species *E. zuernii* and *E. bovis* are generally involved in the highest number of clinical cases in calves and can cause moderate to severe enteritis (Daugschies & Najdrowski, 2005). These animals have abdominal pain, fever, anemia, dehydration, weakness, anorexia (Li et al., 2021) and tenesmus (Daugschies & Najdrowski, 2005). Authors also reports that mortality might reach 7-20% (Pilarczyk et al., 1999). *Eimeria bovis* is one of the main species that produce clinical signals in animals, among which are weight loss, damage to the intestinal endothelium and intermittent hemorrhagic diarrhea (Lopez-Osorio et al., 2020). In the case of *E. bovis* and *E. zuernii* predominate in animals from 4 and 3 months of age (Cruvinel et al., 2018). However, the presence of mixed infections of *E. zuernii* and *E. bovis* has been observed in different ages of animals (Bangoura et al., 2012). This implies that it is not only to observe the presence of Coccidia in the feces, but also to make an identification by species to establish control measures to reduce economic losses in the UPB.

The identification of species and genus through morphological characteristics is controversial because can be subjective and ambiguous between intraspecies variations (Kawahara et al., 2010; Florião et al., 2016). It is mentioned that the size, color and surface of the wall can vary in the ocysts of a given species (Dubey, 2019); therefore, the morphological study is not conclusive and further confirmation by molecular techniques such as PCR is recommended. The presence of specific primers allows molecular identification to be a rapid technique for the diagnosis of pathogenic *Eimeria* spp. (Kawahara et al., 2010). The classification through six specific primers for the pathogenic *Eimeria* species by PCR has been reported (Kawahara et al., 2010; Al-Jubory & Al-Rubaie, 2016; Lee et al., 2018; Ekawasti et al., 2022). Therefore, this aspect requires further research to confirm by PCR *Eimeria* spp. circulating at the UPB in the State of Veracruz, Mexico.

The third objective was to identify the risk factors associated with the presence of *Eimeria* spp. in calves. In this study, different risk factors related to intrinsic (age) and extrinsic factors (*e.g*., physiographic region, presence of other animal species, type of calf rearing and coprological studies) were identified in livestock production units. The 5 - 9-month-old group had a higher number of oocysts compared to 10–13-month-old calves. The prevalence of *Eimeria* natural infection in cattle is highly age dependent (Cornelissen et al., 1995). Young animals up to 1 year of life are usually the most susceptible; also, some studies indicate that this disease is manifested in animals less than 18 months, on average (Makau et al., 2017). As a result, when they grow into adults, they usually become asymptomatic hosts after recurrent reinfections, serving as a source of infection for younger animals (Matjila & Penzhorn, 2002). Lopez-Osorio et al. (2020) found that the most susceptible age to *Eimeria* spp. was between 3 and 6 months, concurring with our findings, where risk was observed in calves between 5 and 9 months of age. The logistic regression analysis revealed an *OR* of 1.49 (CI_95%_ 1.08-2.54; *P* = 0.01), associated with a possible lack of immune response or low immunity (Yun et al., 2000). Therefore, there is a concordance with what has been observed by other authors regarding this factor (Daugschies & Najdrowski, 2005; Almeida et al., 2011; Rehman et al., 2011; Tomczuk et al., 2015). It should be mentioned that although calves obtain immunoglobulins from the colostrum, these are not enough to prevent an infection of the apicomplexans (Faber et al., 2002).

Additionally, the presence of other animal species in the bovine production unit was considered a potential risk factor with an *OR* of 1.86 (CI_95%_ 1.31-2.65; *P*= 0.001) since they facilitate the accumulation of organic matter. Lopez-Osorio et al. (2020) and Rehman et al. (2011) suggest that the lack of cleaning and sanitation, followed by poor sanitation, optimal humidity, concentration of organic matter and temperature are important factors for oocyst sporulation and survival in contaminated soils. Furthermore, the restricted calf suckling was also considered as a risk factor with an *OR* of 2.31 (CI_95%_ 1.37-3.89 and *P=*0.002). In this research, the type of rearing is defined by the way the calves consume the milk, either artificially or directly from the mother (restricted or continuous). Tomczuk et al. (2015) described the highest extent of invasions in those farms where calves were raised with their mothers. Mother ewes have also been found to be the main source of infection for lambs cohabiting with them. Therefore, there is a strong association between possible infected mothers and calves with low immunity who are susceptible to the parasite infection. On the other hand, low *Eimeria* spp. infections were observed in animals that undergo routine coprological studies for the control and treatment of parasites in livestock management. These results agree with the findings of Philippe et al. (2014) who demonstrated that metaphylactic treatment with diclazuril and toltrazuril reduce the impact of coccidiosis by previously identifying the specific parasite to attack. The Papalopan region had an *OR* of 0.50 (CI_95%_ 0.26-0.99; *P=*0.05), suggesting a decrease of the parasite with this variable. The three Municipalities that belong to this physiographic region have dry seasons from September to May approximately, which coincides with the months in which the samples were collected. For an oocyst to be infectious, it must sporulate and mature. An adequate amount of oxygen and moisture is required for oocyst sporulation to occur. If there are periods of drought with constant high temperatures and a decrease in relative humidity, the cycle is interrupted, so the oocyst dies and ceases to be an infectious agent for the calf (Rodríguez-Hawkins, 2011).

## Conclusion

This work revealed the diversity of *Eimeria* species in calves in central region of Veracruz, Mexico. The risk of infection was observed in intrinsic and extrinsic factors, such as age and type of calf rearing. This study will help to implement adequate antiparasitic therapies and management strategies for *Eimeria* spp. infection control, but it also highlights the need to monitor *Eimeria* species considered non-pathogenic in UPBs over time.
